# Proteomic and Metabolomic Signatures of Diet Quality in Young Adults

**DOI:** 10.3390/nu16030429

**Published:** 2024-01-31

**Authors:** Elizabeth Costello, Jesse A. Goodrich, William B. Patterson, Douglas I. Walker, Jiawen (Carmen) Chen, Brittney O. Baumert, Sarah Rock, Frank D. Gilliland, Michael I. Goran, Zhanghua Chen, Tanya L. Alderete, David V. Conti, Lida Chatzi

**Affiliations:** 1 Department of Population and Public Health Sciences, University of Southern California, Los Angeles, CA 90032, USA; jagoodri@usc.edu (J.A.G.); chenjiaw@usc.edu (J.C.); bbaumert@usc.edu (B.O.B.); srock@usc.edu (S.R.); gillilan@usc.edu (F.D.G.); goran@usc.edu (M.I.G.); zhanghuc@usc.edu (Z.C.); dconti@med.usc.edu (D.V.C.); chatzi@usc.edu (L.C.); 2 Department of Integrative Physiology, University of Colorado Boulder, Boulder, CO 80309, USA; william.patterson-1@colorado.edu (W.B.P.); tanya.alderete@colorado.edu (T.L.A.); 3 Gangarosa Department of Environmental Health, Emory University, Atlanta, GA 30329, USA; douglas.walker@emory.edu; 4 Department of Pediatrics, Children’s Hospital Los Angeles, The Saban Research Institute, Los Angeles, CA 90027, USA

**Keywords:** diet quality, metabolomics, proteomics, pathway analysis

## Abstract

The assessment of “omics” signatures may contribute to personalized medicine and precision nutrition. However, the existing literature is still limited in the homogeneity of participants’ characteristics and in limited assessments of integrated omics layers. Our objective was to use post-prandial metabolomics and fasting proteomics to identify biological pathways and functions associated with diet quality in a population of primarily Hispanic young adults. We conducted protein and metabolite-wide association studies and functional pathway analyses to assess the relationships between a priori diet indices, Healthy Eating Index-2015 (HEI) and Dietary Approaches to Stop Hypertension (DASH) diets, and proteins (*n* = 346) and untargeted metabolites (*n* = 23,173), using data from the MetaAIR study (*n* = 154, 61% Hispanic). Analyses were performed for each diet quality index separately, adjusting for demographics and BMI. Five proteins (ACY1, ADH4, AGXT, GSTA1, F7) and six metabolites (undecylenic acid, betaine, hyodeoxycholic acid, stearidonic acid, iprovalicarb, pyracarbolid) were associated with both diets (*p* < 0.05), though none were significant after adjustment for multiple comparisons. Overlapping proteins are involved in lipid and amino acid metabolism and in hemostasis, while overlapping metabolites include amino acid derivatives, bile acids, fatty acids, and pesticides. Enriched biological pathways were involved in macronutrient metabolism, immune function, and oxidative stress. These findings in young Hispanic adults contribute to efforts to develop precision nutrition and medicine for diverse populations.

## 1. Introduction

Recent advances in analytic techniques and “omics” technologies have generated interest in using biomarkers to characterize dietary habits or assess food intake. Omics data include, though are not limited to, the genome, epigenome, transcriptome, proteome, and metabolome and aim to comprehensively characterize all molecules within these domains and construct a holistic view of the mechanisms underlying disease development [1]. In the developing field of precision nutrition, omics methods are currently used to identify potential new biomarkers of dietary intake [2], measure the effects of diet interventions [3], improve upon traditional diet assessments [4], or assess individual responses to dietary interventions [5]. For instance, the metabolome and microbiome have been found to change in response to the Mediterranean diet and other dietary patterns [3,6,7], and proteomic profiles can also reflect dietary habits [8,9,10]. Despite rapid advances in omics technologies and their biomedical and nutritional applications, existing research is limited with respect to the multi-omics integrations and attention to diverse populations that are necessary to bring precision medicine and precision nutrition into clinical practice [11].

Recent improvements in analytical techniques have allowed researchers to characterize the omics phenotypes associated with the consumption of certain foods or the phenotype associated with adherence to healthy dietary patterns, which are usually measured using diet indices such as the Healthy Eating Index 2015 (HEI) [12] and the Dietary Approaches to Stop Hypertension (DASH) diet [13]. Adherence to these healthy eating patterns is associated with a lower risk for diseases like T2D, cardiovascular disease, and other related health outcomes [14,15,16], though the biological processes or mechanisms underlying these effects are not well understood. Proteomics and metabolomics, in particular, are promising methods that may elucidate the molecular foundations of the relationship between diet and disease and have been used to identify pathways of disease development or potential molecular mediators of these associations [15,17].

Though precision medicine and precision nutrition approaches have appeared more frequently in clinical practice and health research [18,19], many populations remain underrepresented. In the United States, Hispanic populations are at increased risk for metabolic-dysfunction associated steatotic liver disease (MASLD) [20], type 2 diabetes (T2D) [21], and other metabolic diseases relative to Non-Hispanic Whites, but are not often included in population-level nutritional or precision medicine investigations [22,23]. The diets of Mexican American and other Hispanic communities in the US have been shown to differ from those of other ethnic groups, with previous work suggesting that Hispanic children and adults may consume different amounts of fast food, sweets, fruit juice, and refined grains [24,25] relative to other ethnic groups. Given that the diet composition may be different in Hispanics, including this population in precision nutrition research is important to ensure equity in future clinical applications.

Despite growing interest in the use of omics technologies to evaluate habitual diet, only a limited number of studies have applied either proteomic or metabolomic assessments to dietary patterns [5,9,10,15,26], and the generalizability of these studies is limited. Of these previous studies, the majority included relatively homogenous populations; a recent review of metabolomics in nutrition found that virtually all took place in North America and Europe, with limited racial or ethnic diversity in the selected populations [26]. The few analyses of dietary patterns and proteomics have also been conducted in limited populations: primarily non-Hispanic White or Black middle-aged and older adults, frequently in the context of characterizing cardiovascular disease risk [9,15]. Young people are also underrepresented, and to date, only two studies have focused on younger adults [10,17]. Additionally, to our knowledge, only one previous study has applied both metabolomics and proteomics methods [27], finding that molecules from both omics layers were associated with each dietary pattern. This study by Walker et al. [27], similar to other omics research, was conducted in middle-aged and older adults from the Framingham Offspring Study, an entirely non-Hispanic White population. Additional research is still needed to assess whether these findings may be generalized to all populations.

The purpose of this study is to use a multi-omics approach to examine the impact of diet quality on biological processes in a population underrepresented in omics research: young adults of primarily Hispanic ethnicity. Our population additionally has a history of overweight or obesity in early adolescence and was previously shown to be at increased risk for type 2 diabetes [28]. Using both proteomics and metabolomics, we will evaluate the associations of two healthy dietary patterns (the HEI and DASH diets) on alterations in the metabolome and proteome to identify molecular signatures of high-quality diet and describe the biological effects of diet using functional pathway analyses.

## 2. Materials and Methods

### 2.1. Study Population

Between 2014 and 2018, 155 participants (aged 17–22) were recruited from the Children’s Health Study in Southern California [29] for the MetaAIR study [30]. To be eligible for the MetaAIR study, participants had to have a history of overweight or obesity in early high school and did not have type 1 or type 2 diabetes. At the clinical visit, participants completed a diet assessment, detailed questionnaires, and an oral glucose tolerance test (OGTT) where plasma was collected. This study was approved by the University of Southern California Institutional Review Board. Written informed consent/assent was obtained from participants or by participants and their guardians for those under age 18.

### 2.2. Diet Assessment

The participants completed two 24 h dietary recalls on non-consecutive days, one weekday and one weekend day, using the Nutritional Data System for Research software version 2014 (University of Minnesota, Minneapolis, MN, USA). Two diet indices, HEI-2015 and DASH, were calculated from the recall data as described previously [28]. HEI-2015 has thirteen components, which are each given a score and summed to a final score between 0 and 100. The components include the following: total fruit, whole fruit, total vegetables, greens and beans, whole grains, dairy, total protein foods, seafood and plant protein, fatty acids, refined grains, sodium, added sugar, and saturated fats [12]. The DASH diet score was calculated from 9 target nutrients (total fat, saturated fat, total protein, fiber, cholesterol, calcium, magnesium, potassium, and sodium), which are each given a value of 0, 0.5, or 1, to generate a final score between 0 and 8 [13]. All components of the HEI and DASH indices, other than fats and protein, were standardized to 1000 kilocalories of intake.

### 2.3. Metabolomics

Untargeted metabolomics were measured in the plasma samples collected after an oral glucose challenge at the two-hour OGTT time point. Liquid chromatography and high-resolution mass spectrometry methods (LC-HRMS) were used as described in Liu et al. [31], with dual column and dual polarity approaches and both positive and negative ionization. This resulted in four analytical configurations: reverse phase (C18) positive, C18 negative, hydrophilic interaction (HILIC) positive, and HILIC negative. Unique features were identified using the mass-to-charge ratio (*m*/*z*), retention time, and peak intensity. The features were adjusted for batch variation [32] and excluded if they were detected in <75% of the samples or if there was a >30% coefficient of variability of the quality control samples after batch correction. After processing, there were 3716 features from the C18 negative mode, 5069 from the C18 positive mode, 7444 from the HILIC negative mode, and 6944 from the HILIC positive mode, for a total of 23,173 features included in the analyses. The raw intensity values from LC-HRMS were scaled to a standard normal distribution and log2 transformed. Details of the analytical process have been described previously [33].

Comparing metabolite features to a database of standards that were analyzed using the same analytical method identified 466 confirmed compounds. Metabolites’ identities were assigned using known standards, and detected peaks were matched using accurate mass *m*/*z* (<5 ppm) and retention time (<15 s). In instances where multiple annotations were possible due to more than one molecule having retention times within the allowable error, the annotation with the closest retention time to the known standard was chosen. Measured *m*/*z* and retention times, theoretical *m*/*z* and retention times, adduct, possible annotations, and additional analytical details are listed in the Supplementary Materials (Table S1).

### 2.4. Proteomics

Proteins were measured in fasting plasma samples using the proximity extension array (PEA) method from Olink Explore 384 Cardiometabolic panel [34]. This panel measures the relative abundance of 369 proteins, reported as normalized protein expression (NPX) levels after log2 transformation [35]. For 23 proteins, 50% of observations were below the limit of detection (LOD) and were excluded from the analysis, leaving 346 proteins from the initial 369 offered after processing.

### 2.5. Covariates

Demographic information was collected through questionnaires. Age was calculated from the visit date and birthday. The participants self-reported race and ethnicity and were categorized as Non-Hispanic White, Hispanic, or Other. Participants’ sex was recorded, and body mass index (BMI, kg/m^2^) was calculated from clinical measurements of weight and height.

### 2.6. Statistical Analysis

Descriptive statistics were calculated for all diet indices and covariates, including mean and standard deviation for continuous variables and frequency and percent for categorical variables. Descriptive statistics were also calculated for participants above and below the median for HEI and DASH.

**Omics-Wide Association Studies (OWAS).** Protein association studies (PASs) and metabolite-wide association studies (MWASs) were conducted between the diet indices and each of the 346 proteins and 23,173 metabolite features individually using linear regression models. To account for multiple testing, we also report *p*-values adjusted for a false discovery rate (FDR) (*q*-values), calculated using the Benjamini–Hochberg method [36]. Q-values of <0.05 were considered additional evidence for a true association between diet and that individual protein or metabolite. Each linear model was adjusted for age, sex, ethnicity, and BMI. Energy was not included as a covariate as both diet quality indices had already standardized each component to kilocalorie intake. All analyses were performed using R, version 4.1.0 (R Foundation for Statistical Computing, Vienna, Austria).

**Pathway Analysis.** Proteomic pathway enrichment analysis was conducted using the core analysis function of Qiagen Ingenuity Pathway Analysis (IPA) separately for each diet quality index (QIAGEN Inc., https://digitalinsights.qiagen.com/IPA, accessed on 31 October 2023) [37]. To identify pathways associated with either DASH or HEI, UniProt identifiers and test statistics (t-scores) for proteins significant in the PAS analysis at *p* < 0.05 were uploaded to IPA separately for each diet quality index. Canonical pathways associated (*p* < 0.05) with each diet quality index, which included three or more protein hits, were extracted. A metabolomic pathway enrichment analysis was performed for each diet quality index using the effect estimates and t-scores for each diet index-metabolite relationship. The analysis was conducted using MetaboAnalyst (version 5.0), version 2.0 of the MS peaks to path module [38], using the mixed ion mode, 5 ppm mass tolerance, and Human MFN pathway library, with a *p*-value threshold of 0.05. Effect estimates (β), t-scores, and *p*-values for each diet–metabolite relationship in the MWAS analysis were uploaded to MetaboAnalyst (https://www.metaboanalyst.ca/, accessed on 4 April 2022). Only pathways with three or more metabolite hits were extracted. Integrated Gene Set Enrichment Analysis (GSEA) [39] and mummichog [40] algorithms were used to identify significantly enriched pathways (*p* < 0.05) based on the combined *p*-values from both methods [41].

**Sensitivity Analysis.** To assess the impact of further adjustment for energy intake, a sensitivity analysis was performed with PAS and MWAS, adding daily kilocalorie intake as a covariate.

## 3. Results

### 3.1. Study Population Characteristics

At the study visit, the average age of participants was 19.7 years (SD: 1.2), and 61% were of Hispanic or Latino ethnicity (Table 1). The participant demographics were similar in those with high (above or equal to the median) and low (below the median) diet quality scores, except for age and energy intake, which were higher in those with below-median DASH scores. The participants had a mean HEI score of 52.7 (SD: 13.0) out of 100 and a mean DASH score of 2.3 (1.5) out of 8. HEI and DASH scores were significantly positively correlated (r = 0.45, *p* < 0.001). Of the 155 MetaAIR participants with complete diet assessment data, 1 was missing proteomics data, and 31 were missing metabolomics, leaving 154 and 124 with complete data for proteomics and metabolomics analyses, respectively.

### 3.2. Diet Quality Was Associated with Proteins and Metabolites

**Figure 1:**
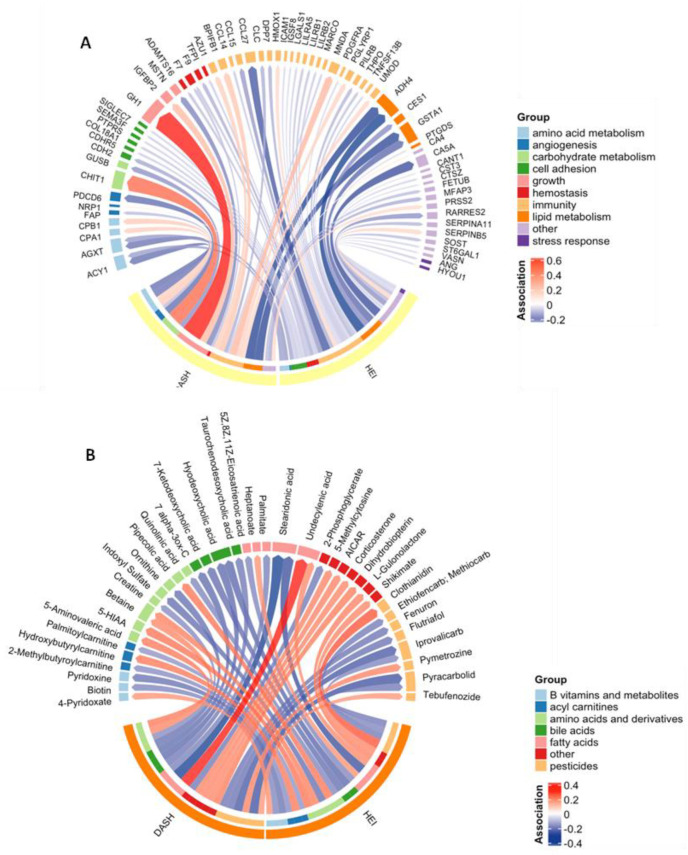
Chord diagram showing the significant (*p* < 0.05) associations between each diet quality index and each of the proteins (**A**) and annotated metabolites (**B**). The width of each arrow represents the magnitude of the association, scaled to 1 standard deviation of exposure, and the color represents the direction. Proteins and metabolites are grouped by function. Abbreviations: 5-hydroxyindoleacetic acid (5-HIAA); 5-aminoimidazole-4-carboxamide ribonucleotide (AICAR).

PAS identified 44 proteins associated with HEI and 25 associated with the DASH score at a *p*-value threshold of 0.05 (Figure 1A). This represented 64 (17.5%) unique features associated with at least one diet quality index. There were five features (ACY1, ADH4, AGXT, F7, and GSTA1) associated with both diet indices (Table S2). These proteins were all inversely associated with HEI and DASH and were involved in amino acid metabolism, immunity, lipid metabolism, and hemostasis. No proteins were statistically significantly associated with either diet quality index after adjustment for multiple comparisons (*q* < 0.05).

MWAS was performed on all 23,173 untargeted metabolomic features to examine their associations with each diet quality index. We found that 1250 metabolomic features were associated with the HEI, and 1106 were associated with DASH scores (Table S3). Of the 2101 (9.0%) unique features associated with either diet quality index at a *p*-value threshold of 0.05, 38 had confirmed annotations (Figure 1B, Table S4). These metabolites included compounds belonging to groups such as amino acids and components of amino acid metabolism, fatty acids, bile acids, and pesticides. Six annotated metabolites were associated with both diet indices: undecylenic acid (a medium-chain fatty acid) and betaine (an amino acid derivative) were positively associated with HEI and DASH, while iprovalicarb and pyracarbolid (fungicides), stearidonic acid (an omega-3 fatty acid and derivative of lineolic acid), and hyodeoxychoic acid (a bile acid) were inversely associated with HEI and DASH. After adjustment for multiple comparisons (*q* < 0.05), five features were associated with DASH (Table S3). None of these features had confirmed annotations.

After further adjustment for energy intake in the sensitivity analysis, 42 proteins were associated with HEI, and 26 were associated with DASH (*p* < 0.05) (Table S5), representing 62 unique proteins. Six were associated with both diet indices: ACY1, F7, GSTA1, HMOX1, NECTIN2, and RARRES2. No proteins were associated with either diet quality index after adjustment for multiple comparisons (*q* < 0.05).

MWAS results further differed after energy adjustment: 1563 proteins were associated with HEI, and 1236 were associated with DASH (Table S6). Of these, twenty-eight had confirmed annotations and six were associated with both diet indices: *n*-formylglycine, 2-methyl-4-pentenoic acid, indole-3-aldehyde, methyl jasmonate, 5-deoxyadenosine, and ipconazole (Table S7). After adjustment for multiple comparisons (*q* < 0.05), no metabolomic features were associated with HEI and one unannotated feature was associated with DASH (Table S6).

### 3.3. Diet Quality Was Associated with Biological Pathways

**Figure 2:**
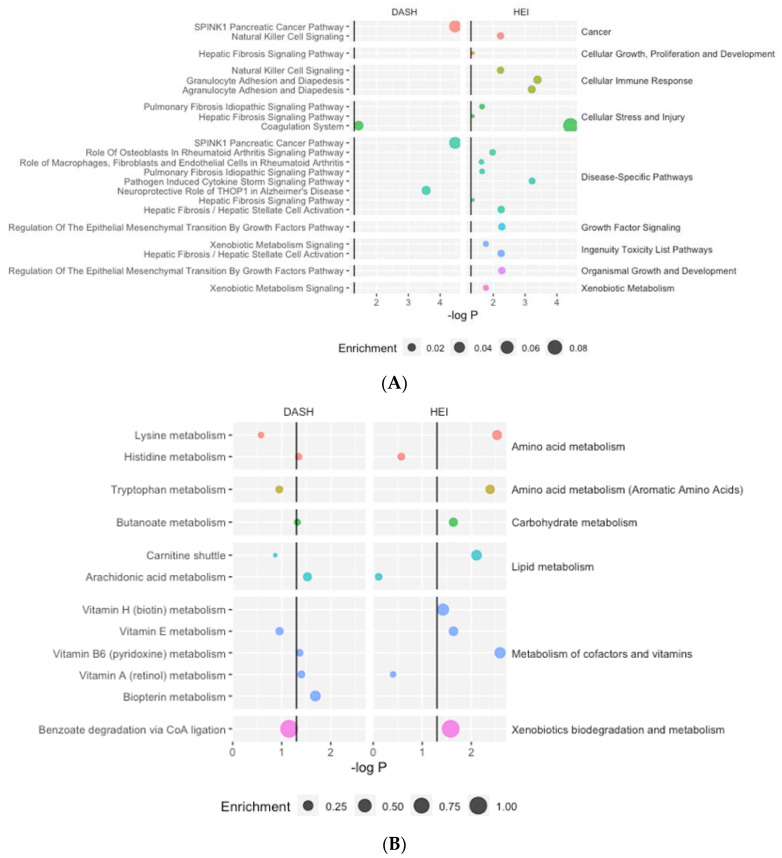
Significantly enriched proteomics (**A**) and metabolomics (**B**) pathways. Enrichment refers to the number of significant features within the pathway divided by the total number of features in the pathway.

The results from the proteomics core analysis in IPA identified 14 unique canonical pathways associated with at least one diet quality index (Figure 2A, Table S8). HEI was associated with twelve pathways, and DASH was associated with two pathways.

Pathway analysis identified twelve unique metabolic pathways associated with at least one diet quality index, with HEI associated with eight significantly enriched pathways and DASH with six significantly enriched pathways (Figure 2B, Table S8). Most pathways were related to amino acid metabolism and the metabolism of cofactors and vitamins. Two pathways (butanoate metabolism and vitamin B6 metabolism) were significantly enriched for both diet quality indices.

## 4. Discussion

In a population of primarily Hispanic young adults with a history of overweight or obesity, we identified possible metabolomic and proteomic signatures of a high-quality diet, as measured using two established dietary indices: HEI-2015 and the DASH diet. We identified five proteins (F7, ACY1, ADH4, AGXT, and GSTA1) and six metabolites (betaine, iprovalicarb, pyracarbolid, undecylenic acid, stearidonic acid, and hyodeoxycholic acid) with inverse associations with both the HEI and DASH diets, and two metabolites (betaine and undecylenic acid) positively associated with both diets. Pathway analysis indicated that adherence to these diets was associated with liver function, immune response, hemostasis, and multiple disease-specific pathways. The results from both proteomics and metabolomics analyses revealed omics signatures involved in amino acid and lipid metabolism, as well as oxidative stress and inflammation, consistent with previous evidence in older adults and non-Hispanic populations [27,42]. However, the specific proteins and metabolites we identified in this study have not been previously reported as linked to diet quality and may represent novel biomarkers in our specific population.

Many of the proteomic and metabolomic features identified in our study have been previously linked to dietary intake, metabolic disease, or both. Higher levels of the protein F7 (coagulation factor VII) have been linked to cardiovascular disease and obesity [43], while previous research indicates that proteins ACY1 (aminocyclase 1) and GSTA1 (glutathione S-transferase alpha 1) are positively associated with T2D [44,45]. Circulating levels of betaine, a compound involved in homocysteine metabolism and carnitine production, has frequently been positively associated with reduced risk for T2D [46], a healthy gut microbiome [47], and lower risk for other metabolic and cardiovascular diseases [48]. Undecylenic acid has also been identified as a possible indicator of gut microbiome health [49] and has been found to have anti-inflammatory and antioxidative properties [50,51]. Additionally, the functional pathways identified in this analysis are consistent with the known antioxidant, anti-inflammatory, and immune responses associated with healthy diets [52]. Both the HEI and the DASH diets are characterized by high intake of fiber, whole grains, fruit and vegetables, and low intake of sodium, added sugar, and saturated fats, all dietary components associated with beneficial circulating proteins and metabolites [27].

Our analysis also identified some proteins and metabolites associated with diet that have not previously been specifically linked to dietary intake or metabolic disease. For instance, ADH4 (alpha dehydrogenase 4), a protein involved in alcohol oxidation [53], is not known to be associated with diet, though it is involved in retinol metabolism [54]. AGXT (alanine-glyoxylate aminotransferase) is a liver enzyme responsible for the oxidation of glyoxylate to oxalate, as well as a catalyst for aldehyde–ketone and amino acid interconversions [55]. Though circulating AGXT has not been previously reported as a biomarker of diet or metabolic disease, impaired expression of the AGXT gene may contribute to the development of atherosclerosis [56], and AGXT in liver tissue may be a biomarker for hepatocellular carcinoma [57].

Several unexpected metabolites were also identified. Stearidonic acid was inversely associated with DASH and HEI in our analysis, and although it may appear unusual that an omega-3 fatty acid is inversely associated with a healthy diet, because stearidonic acid is not usually a major component of the diet and is rapidly converted to eicosapentaenoic acid (EPA), low levels may indicate that more EPA is being generated [58,59]. Hyodeoxycholic acid is a secondary bile acid produced by gut microbiota and is involved in the regulation of lipid homeostasis [60]. High levels of hyodeoxycholic acid and other bile acids are a possible sign of diet-related liver disease, though existing research is limited [61]. Additionally, HEI and DASH were positively associated with some pesticide metabolites (tebufenozide, pymetrozine, and clothianidin) and inversely associated with others (ethioencarb/methiocarb, fenuron, flutriafol, iprovalicarb, and pyracarbolid). The human health effects, if any, of these specific compounds are not clear, and there are few reports of their detection in human tissue.

In our population, both HEI and DASH were associated with proteins involved in amino acid and lipid metabolism, immune system function, and hemostasis. Both diets were also associated with fatty acid, amino acid, bile acid, and pesticide metabolites and with similar metabolic pathways. This is likely due to similarities between the diet quality indices, each of which rewards increased consumption of fiber and unsaturated fatty acids and penalizes consumption of sodium and saturated fat. These nutrients have previously been linked to fatty acid metabolism [62], gut microbial composition [63], and immune function [64]. However, each dietary pattern was also distinguished by unique associations with the features and functions of specific omics. HEI was associated with B vitamin and acyl carnitine metabolites, proteins related to stress response and cell adhesion, and proteomics pathways related to cellular immune response, cell and organismal growth and development, and multiple disease pathways. In contrast, only DASH was associated with proteins involved in angiogenesis, and few proteomics pathways were significantly enriched in response to the DASH diet. These differences may be due to the different components used to calculate each index. The HEI includes components from whole foods, such as fruits, vegetables, whole grains, and protein foods. These components include likely sources of B vitamins (beans, green vegetables, animal protein) [65], foods that contain antioxidants or anti-inflammatories (fruits, vegetables) [66], and food groups that may reduce the risk of disease (fruit, vegetables, dairy, and whole grains) [67]. Because our measure of the DASH diet includes only nutrients and no whole foods, this score may not have captured these additional biomarkers and pathways.

Our study population represents a specific subgroup of young people at increased risk for diet-related metabolic disease: scores on the HEI and DASH are relatively low, participants had a history of overweight or obesity in adolescence, with many continuing to have a BMI in the overweight or obesity categories, and Hispanic ancestry is an additional risk factor for cardiometabolic disease [68]. The relationships between the metabolites and proteins identified here and macronutrient metabolism, inflammation, oxidative stress, gut microbial activity, and risk for specific metabolic conditions confirm that these are potential candidates to track adherence to dietary interventions or developing risk for disease. The results of this analysis may contribute to “precision medicine” approaches, eventually allowing treatments or interventions to be tailored to individuals’ specific biological states [69]. This study is one of the first to assess the combination of both proteomics and metabolomics, which may provide more information about an individual’s diet than each omics layer alone. For instance, circulating proteins may reflect long-term or habitual diet, while metabolomics may be more responsive to recent intake or to specific foods as well as long-term dietary intake [8,70]. However, our results do not perfectly match those of the only other study to examine dietary patterns and proteomic and metabolomic profiles; we did not identify the same individual proteins and metabolites as Walker et al. [27], though overall molecular functions and biological pathways were similar. These differences may be related to differences in our study population, which was much younger, had a higher average BMI, and was more than 50% Hispanic, or differences in measured proteins and metabolites. Additional studies are needed to validate both our and previous findings and ensure that diverse populations are represented.

This study has multiple strengths, including the use of untargeted post-prandial metabolomics and a large proteomics panel, which provided over 800 identified proteins and metabolites. Metabolomics quantified after a standardized intervention, such as a glucose challenge, may provide more information on overall metabolic status than metabolomics measured in fasting samples [71]. The use of two established diet quality indices allowed us to identify features consistent across different measures of diet quality, while analyzing both proteomics and metabolomics allowed us to assess biological processes and pathways impacted by both omics layers. This analysis, conducted in a population of primarily Hispanic young adults, may also reflect dietary intake or omics signatures specific to this age or ethnic group or biological effects unique to young people with a history of high BMI. Our study participants come primarily from Hispanic and Latinx communities in Southern California, and these communities have been understudied in precision nutrition research.

Limitations include the relatively small sample size, which likely reduced our ability to detect statistically significant features. However, our findings are biologically plausible, and the biological pathways and functions we identified are similar to those described in previous work. By including two diet quality indices and performing pathway analyses, we are able to show that biological effects are similar across diet quality indices, omics layers, and previous studies, though we have few associations that are statistically significant after adjustment for multiple testing. Despite this, there remains inconsistency in the diet–omics findings between our work and previous studies; as the complexity of omics analyses increases, additional methods will be needed to determine the extent to which inconsistencies are due to population differences, measurement methods, or noise. We also observed that further adjustment for kilocalories produced somewhat different results from unadjusted analyses, and careful consideration may be required to determine which level of energy adjustment is most appropriate for omics analyses. Finally, this cross-sectional study reports associations between diet and omics measurements collected at a single time point. It is possible that this diet assessment did not completely capture habitual intake, which may attenuate our observed effects.

## 5. Conclusions

Our study identified proteins, metabolites, and biological pathways associated with a healthy diet, as measured using HEI and DASH diet indices, in a population of predominately Hispanic young adults with a history of overweight or obesity. Five proteins (ACY1, ADH4, AGXT, GSTA1, F7), and six metabolites (undecylenic acid, betaine, hyodeoxycholic acid, stearidonic acid, iprovalicarb, pyracarbolid) were associated with both HEI and DASH. Several of these features, including F7, ACY1, GSTA1, betaine, and undecylenic acid, have previously been linked to cardiometabolic disease. These results, which included features and pathways linked to amino acid metabolism, the gut microbiome, immune function, oxidative stress, and inflammation, emphasize the importance of a healthy diet on metabolic function and suggest that there is potential for proteomics and metabolomics measurements to play a role in clinical assessments and interventions. Our work contributes to efforts to identify biomarkers of a healthy diet that may be involved in the development of metabolic disease in diverse populations.

## Figures and Tables

**Table 1:** Demographics for all participants and for participants stratified by diet quality scores above and below the medians.

Variable	All Subjects *n* = 155	HEI			DASH		
		<51.6 (*n* = 77)	≥51.6 (*n* = 78)	*p*-Value	<2 (*n* = 66)	≥2 (*n* = 89)	*p*-Value
Age (years), Mean (SD)	19.7 (1.2)	19.8 (1.3)	19.7 (1.1)	0.64	20.1 (1.3)	19.5 (1.1)	0.004
Sex, *n* (%) Female Male	71 (45.8) 84 (54.2)	31 (39.7) 47 (60.3)	40 (51.9) 37 (48.1)	0.13	33 (50.0) 33 (50.0)	38 (42.7) 51 (57.3)	0.37
Ethnicity, *n* (%) Hispanic/Latino Non-Hispanic White Other	94 (60.6) 52 (33.5) 9 (5.8)	42 (53.8) 31 (39.7) 5 (6.4)	53 (68.8) 21 (27.3) 3 (3.9)	0.16	38 (57.6) 24 (36.4) 4 (6.1)	57 (64.0) 28 (31.5) 4 (4.5)	0.70
BMI (kg/m^2^), Mean (SD)	29.9 (5.1)	30.1 (5.0)	29.6 (5.2)	0.49	29.9 (4.6)	29.8 (5.5)	0.89
Energy Intake (kcal), Mean (SD)	2050 (630)	2110 (629)	1990 (628)	0.23	2260 (561)	1900 (638)	<0.001
HEI, Mean (SD)	52.7 (13.0)						
DASH, Mean (SD)	2.26 (1.51)						

Abbreviations: HEI, Healthy Eating Index 2015; DASH, Dietary Approaches to Stop Hypertention; BMI, body mass index; SD, standard deviation. *p*-values represent tests for difference using two-sides *t*-tests (continuous variables), Chi-square tests (sex), and Fisher’s exact tests (ethnicity).

## Data Availability

The data presented in this study are available upon request from the corresponding author. The data are not publicly available to protect participants’ identifiable information.

## Supplementary Materials

The following supporting information can be downloaded at: https://www.mdpi.com/article/10.3390/nu16030429/s1, Table S1. Annotated metabolites analytical details; Table S2: Protein association study results; Table S3: MWAS results for all untargeted features; Table S4: Annotated metabolites associated (*p* < 0.05) with either HEI or DASH; Table S5: Protein association study results after adjustment for kilocalories; Table S6: Untargeted metabolite-wide association study results after adjustment for kilocalories; Table S7: Annotated metabolites significantly associated with either HEI or DASH after adjustment for kilocalories; Table S8: Proteomic and metabolomic pathways and their contributing features.
